# Using the Rasch measurement theory to assess the psychometric properties of the Hopkins Symptom Checklist-10 in adolescents

**DOI:** 10.1186/s12955-021-01884-9

**Published:** 2021-10-27

**Authors:** Hanne Søberg Finbråten, Annette Løvheim Kleppang, Anne Mari Steigen

**Affiliations:** 1 Department of Health and Nursing Sciences, Faculty of Social and Health Sciences, Inland Norway University of Applied Sciences, PO Box 400, 2418 Elverum, Norway; 2 Department of Public Health and Sport Sciences, Faculty of Social and Health Sciences, Inland Norway University of Applied Sciences, PO Box 400, 2418 Elverum, Norway

**Keywords:** Adolescents, Hopkins Symptom Checklist, Psychological distress, Psychometrics, Rasch measurement theory, Validation

## Abstract

**Background:**

The Hopkins Symptom Checklist-10 (HSCL-10) is widely used to measure psychological distress in adolescents. To provide valid and reliable results and generate recommendations for practice and policies, instruments with sound psychometric properties are required. The purpose of this study is to use Rasch measurement theory to assess the psychometric properties of the HSCL-10 among adolescents aged 13–19.

**Methods:**

In this cross-sectional study, 6445 adolescents responded to a web-based questionnaire. Data were collected from lower and upper secondary schools in Norway during 2018. The data were analysed using the partial credit parameterisation of the unidimensional Rasch model.

**Results:**

HSCL-10 was found to be unidimensional and to have acceptable reliability. One pair of items showed response dependency. The targeting of the instrument could have been better. All items had ordered thresholds. Three items under-discriminated and three displayed differential item functioning regarding gender.

**Conclusions:**

HSCL-10 has potential for measuring psychological distress in adolescents, though there is room for improvement. To further improve this instrument, some items should be rephrased.

## Background

Mental health problems among adolescents are a leading cause of disease burden worldwide [1] and an important public health issue in Norway and other Western countries. Studies have reported an increase in health complaints and mental health problems among young individuals over the last decades [2–4], with girls have reporting higher psychological distress than boys [5, 6]. Hence, it is important to identify those at risk of mental health problems to provide adequate services.

In epidemiological studies, questionnaires measuring depression and anxiety symptoms are frequently used to collect self-reported data about psychological distress. Several instruments have been developed for this purpose, such as the Hopkins Symptom Checklist (HSCL) [7, 8], Hospital Anxiety and Depression Scale [9] and the General Health Questionnaire [10].

Hopkins Symptom Checklist-10 (HSCL-10) is commonly used to measure psychological distress in many Western countries [8, 11, 12]. An early version of HSCL was developed in the 1950s [7] but has since undergone several revisions [8]. HSCL was originally intended to examine the efficacy of psychotropic drugs and contained questions pertaining to symptomatic behaviour of outpatients in adult populations [8]. This instrument is available in different versions of different lengths (5–90 items). Among young individuals, the short versions primarily have been used (5, 10 and 25 items). Earlier studies have shown that HSCL-10 is suitable for the identification of psychological distress in adolescents [13–15].

Generally, measurements with sound psychometric properties are important for clinical and research purposes [16]. Previous psychometric examinations of HSCL instruments mainly included Cronbach’s alpha coefficient and factor analysis. One study has examined the psychometric properties of HSCL-10 using Rasch measurement theory (RMT) among Norwegian adolescents aged 15–16 [14]. However, this study was based on data from 2001 and 2009, and the analyses were based on data from only one region of Norway. Another recent study among adolescents in Norway assessed the psychometric properties of a six-item depressive symptom scale that includes five items from the HSCL-10 [17]. Generally, the Rasch model facilitates the disclosure of measurement problems that may not be easily detected by traditional analyses, such as lack of invariance, commonly called differential item functioning (DIF). As adolescents’ mental health may have changed over the last decade and there might be differences in how well the instrument is suited for adolescents at different ages, there is a need to update knowledge about the psychometric properties of the HSCL-10. Therefore, the aim of this study is to use RMT to assess the psychometric properties of HSCL-10 among adolescents aged 13–19.

## Methods

### Data collection and study population

This study was based on Ungdata from 2018. Ungdata is a Norwegian national cross-sectional survey collecting data annually among adolescents in lower and upper secondary schools. The results from the Ungdata survey are frequently reported in the media and used when providing recommendations for practice and policies. All data collection is performed by Norwegian Social Research at Oslo Metropolitan University, in cooperation with all regional drug and alcohol competence centres. This survey is partially financed by the Norwegian Directorate of Health. All participants completed an anonymous web-based questionnaire at school. A teacher was present during data collection to help the participants if they had any questions. All parents were informed about the study in advance. Parents of adolescents aged 13–17 were informed that they can withdraw their children from the study at any time. All adolescents were informed that participation was voluntary. This study was ethically approved by the Norwegian Centre for Research Data [18].

Ungdata covers areas such as mental and physical health, relationships with peers and parents, drug use, leisure time activities and nutrition. The survey consists of a main module that all the respondents are supposed to answer, along with optional modules that the municipality has an opportunity to choose. Some of the items of the HSCL-10 are included in the main module, whereas others only in the optional one.

In this study, 6445 participants, aged 13–19, who answered all the HSCL-10 questions, were included. The analysis was, therefore, based on complete data. Six of the questions were from the main module, and four were from one of the optional modules.

In total, 73% of the respondents (aged 13–16) were recruited from lower secondary schools (Table 1). The sample comprised an approximately equal proportion of males and females.

**Table 1:** Sample characteristics (*n* = 6445) of participants in the Ungdata survey, 2018

Characteristic	*n* (%)
Gender	
Male	3166 (49.1)
Female	3255 (50.5)
Missing	24 (0.4)
Education	
Lower secondary school	4709 (73.1)
Grade 8	1553 (33.0)
Grade 9	1525 (32.4)
Grade 10	1495 (31.8)
Missing	136 (2.9)
Upper secondary school	1736 (26.9)
Year 1	733 (42.2)
Year 2	604 (34.8)
Year 3	399 (23.0)
Missing	0

### Hopkins Symptom Checklist-10

HSCL-10 is a short version of the Hopkins Symptom Checklist-25 that has been developed to measure symptoms of anxiety and depression [8, 19]. It consists of 10 items in which adolescents are asked whether during the previous week they had any symptoms of anxiety or depression (Table 2). All items have four response categories: ‘Not been affected at all’ (1), ‘Not been affected much’ (2), ‘Been affected quite a lot’ (3) and ‘Been affected a great deal’ (4). Higher scores indicate higher levels of psychological distress [19].

**Table 2:** Item fit statistics for the Hopkins Symptoms Checklist-10 (*n* = 6445)

Item	Label	Location	Fit residual	χ^2^	Probability*
1	Suddenly felt scared for no reason	0.845	− 1.642	31.474	< 0.001
2	Felt constant fear and anxiety	0.952	− 7.010	85.170	< 0.001
3	Felt exhausted or dizzy	0.234	4.650	25.485	< 0.001
4	Felt stiff or tense	− 0.251	4.729	26.004	< 0.001
5	Tended to blame yourself for things	0.049	− 4.440	68.399	< 0.001
6	Had sleep problems	− 0.480	12.204	142.771	< 0.001
7	Felt unhappy, sad or depressed	− 0.343	− 12.539	214.455	< 0.001
8	Felt worthlessness	0.132	− 4.802	59.194	< 0.001
9	Felt that everything is a struggle	− 0.946	− 4.067	38.367	< 0.001
10	Felt hopelessness about the future	− 0.192	− 3.443	40.011	< 0.001

*Bonferroni-adjusted 5%

### Rasch measurement theory

In this study, the psychometric properties of HSCL-10 were analysed using the partial credit parameterisation [20] of the unidimensional Rasch model [21]. If data from an instrument fit the unidimensional Rasch model, it is statistically defensible to sum the responses to each single item to a total score for each person [16]. At a general level, dimensionality, response dependency and targeting were examined. Dimensionality was examined using *t*-tests on person estimates of items intended to measure depression and anxiety symptoms. A residual correlation between two items of < 0.3 was used as an indicator of response dependency between the two items (i.e. the answer of one item is dependent on the answer of another). In addition, residual correlations of items were assessed relative to each other [22].

Reliability was assessed using the Person Separation Index (PSI), which is based on non-linear transformation of raw scores and indicates a scale’s ability to differentiate individuals along the latent trait. The PSI is analogous to Cronbach’s alpha [23]. Targeting indicates how well a scale captures the person estimates. A scale is considered well targeted if the mean person location values are around zero [24].

Analyses at a finer level included item fit, ordering of response categories and DIF. Chi-square statistics and standardised residuals based on comparisons between observed and expected values were used to analyse item fit. Chi-square probability values above Bonferroni’s adjusted 5% and fit residuals in the range ± 2.5 indicate adequate item fit [24]. Item characteristic curves (ICCs) were inspected to assess item fit graphically.

Significance tests like chi-square are sensitive to sample size. Since this study included a rather large sample size, there was a risk of drawing false conclusions [25]. The amend sample size function in RUMM was, therefore, used to draw a random sub-sample for further analyses concerning item fit and DIF. As recommended, sample size is calculated by multiplying the number of items (10) by the number of thresholds (3) with 30 persons per threshold [26], yielding a sample size of 900 (10 × 3 × 30), which can be deemed as adequate in these analyses.

A central requirement of measurement is that every item should work invariantly across levels of different person factors, such as gender and school level. To examine DIF, two-way analysis of variance of standardised residuals was used [27]. Statistical significance was assumed at a Bonferroni-adjusted 5%, and graphical displays (i.e. ICCs) were used. DIF analyses were performed for the person factors gender, school level and grade. DIF can be handled either by resolving, deleting the item, or ignoring the DIF [28]. Resolving DIF implies splitting the item into e.g. gender-specific items and treating the opposite person factor category as a non-response. To distinguish real from artificial DIF, the items were sequentially resolved, starting with the item having the highest F-value [29]. Additionally, the mean person estimates, chi-square values and PSI were compared before and after splitting the items [26, 30]. Response categories were found to be ordered if the thresholds were significantly different and in the right order [23]. All analyses were performed using the software RUMM2030Plus [31], that handles missing data through FIML (full information maximum likelihood).

## Results

The HSCL-10 items formed a unidimensional scale (the proportion of significant *t*-tests of the difference in person–location estimates between subsets of items was 3.48%) with acceptable reliability (PSI = 0.823, Cronbach’s alpha = 0.913). Items 1 and 2 showed evidence of response dependency (residual correlation = 0.42). Four other pairs of items also showed a positive residual correlation (items 7 and 8, 3 and 5, 5 and 8, and 7 and 10), but the value was 0.1 or lower. All other residual correlations were negative.

**Fig. 1:**
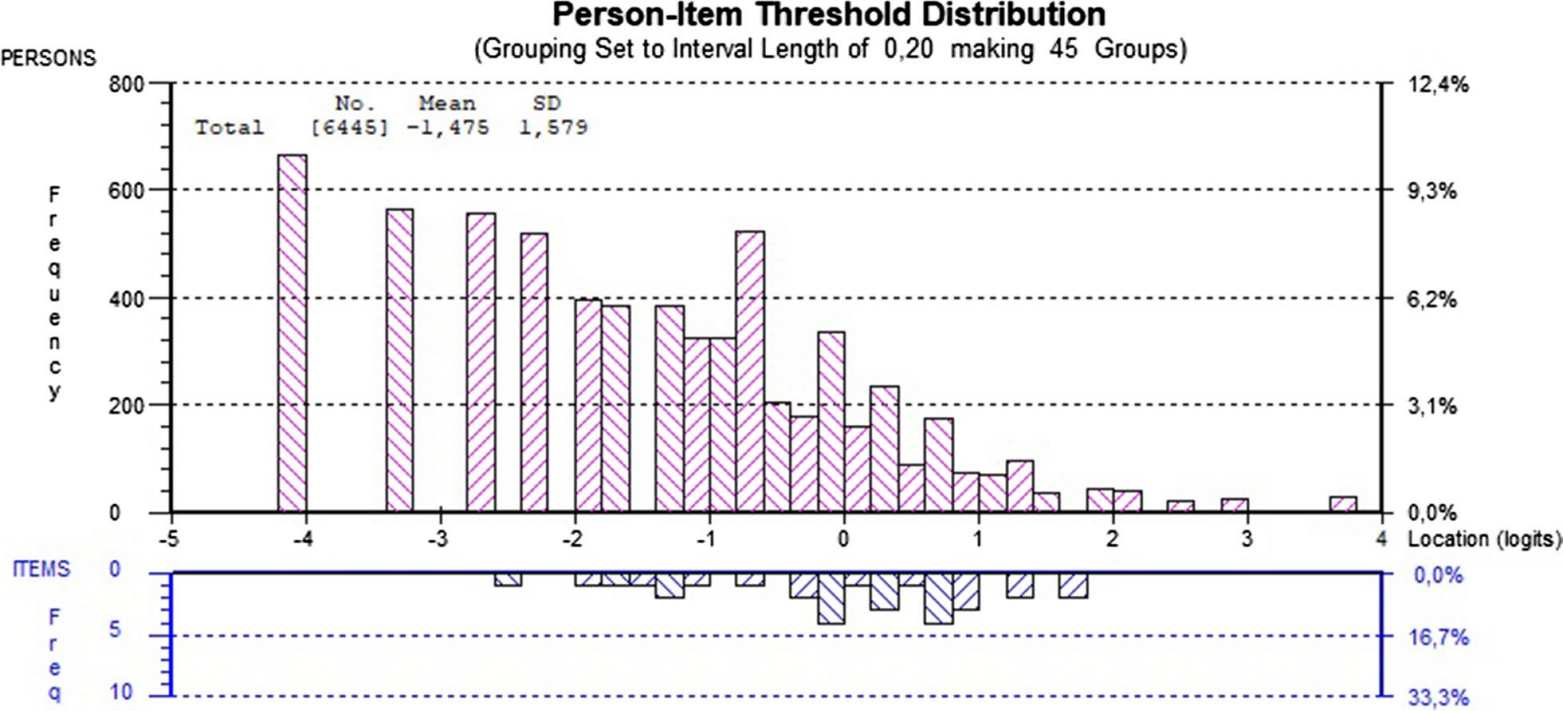
Distribution of person estimates (above the x-axis) and item threshold estimates (below the x-axis). The person estimates indicate that the adolescents (n = 6445) have lesser psychological distress than captured by the Hopkins Symptom Checklist-10

Comparing the distribution of person estimates to the item threshold estimates centred around zero revealed that the person thresholds showed a skewed distribution, with the main weight on the left. Hence, the instrument can be considered somewhat out of target as the mean person location was − 1.475 (Fig. 1). The person–item threshold distribution also indicated that the instrument was better targeted at females (mean value =  − 0.937) than at males (mean value =  − 2.029). The targeting was also better for adolescents in upper secondary schools (mean value =  − 1.241) than for those in lower secondary schools (mean value =  − 1.561).

### Item fit

Given the sample size, statistically significant chi-square values were observed for all items (Table 2).

**Fig. 2:**
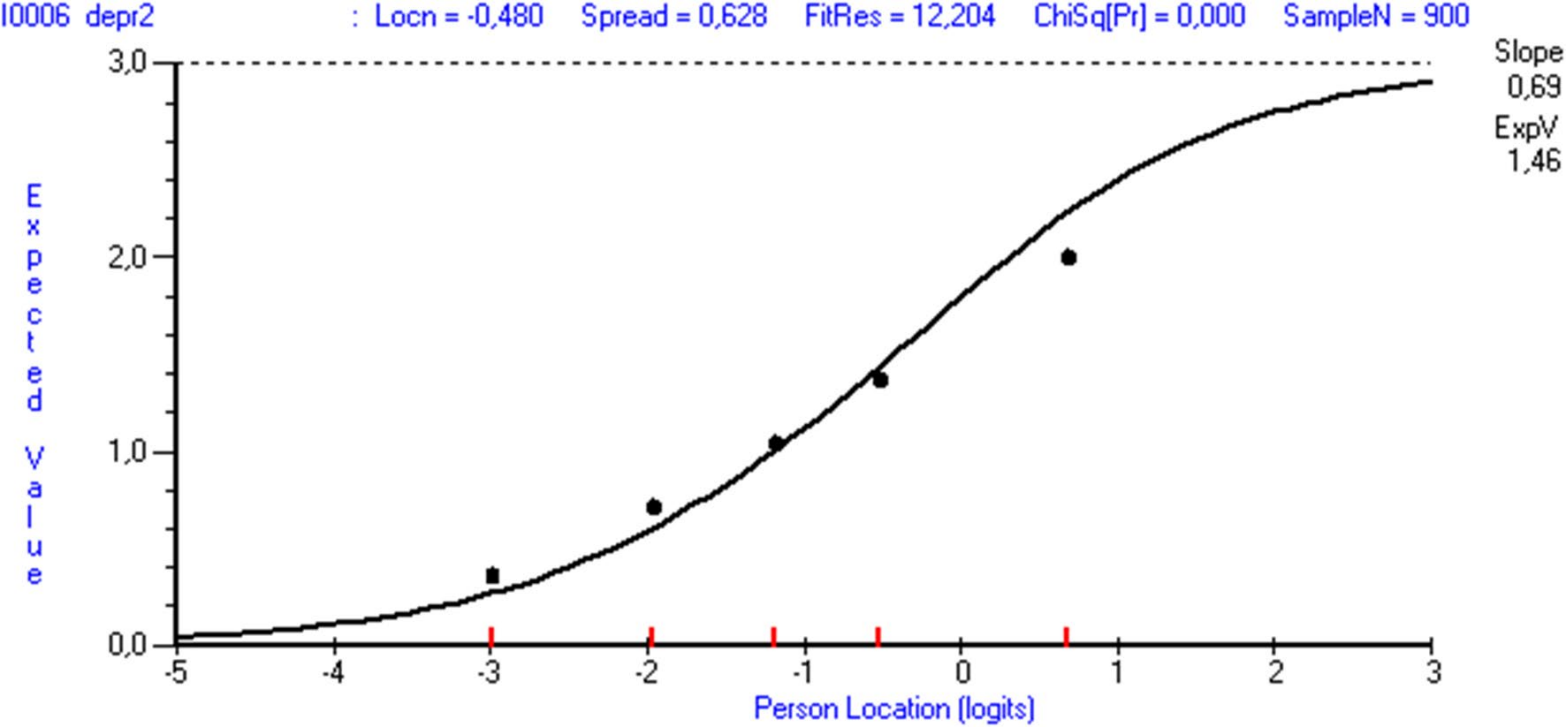
Item-characteristic curve for item 6 in Ungdata 2018 (reduced sample, *n* = 900). *ChiSq[Pr]* Chi Square probability, *FitRes* fit residual: the difference between the response to an item and the expected value according to the model, *Locn* location, item location estimate, *Spread* spread of the thresholds

Three items under-discriminated (items 3, 4 and 6), and seven over-discriminated (items 2, 5, 7, 8, 9 and 10; Table 2). When the sample size was reduced to 900, only items 6 and 7 had significant chi-square values. This was also valid when analysing respondents from lower and upper secondary schools separately. The graphical presentation of the observed compared to the expected values for item 6 shows that the curve is flatter than expected and that the item tends to under-discriminate (Fig. 2).

For all items the thresholds separating the categories were significantly different and in the correct order, indicating that the response categories worked well (Table 3).

**Table 3:** Uncentralised thresholds for each of the Hopkins Symptoms Checklist-10 items

Item	UnCThr 1	UnCThr 2	UnCThr 3
1	0.079	0.821	1.634
2	0.375	0.788	1.693
3	− 1.282	0.603	1.382
4	− 1.598	− 0.063	0.908
5	− 1.058	− 0.038	1.244
6	− 1.842	− 0.269	0.672
7	− 1.741	− 0.104	0.815
8	− 0.338	0.265	0.469
9	− 2.592	− 0.604	0.357
10	− 1.236	− 0.012	0.672

*UnCThr* uncentralised threshold

### Differential item functioning

**Fig. 3:**
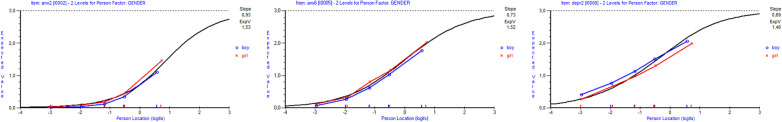
Expected value curves for items 2, 5 and 6 divided by gender. Based on reduced sample, *n* = 900

When the sample size was reduced to 900, three items (items 2, 5 and 6) displayed DIF regarding gender. Item 6 had the greatest magnitude of gender DIF (F-value = 18.7). Figure 3 shows the DIF for the person factor gender for items 2, 5 and 6.

Females tended to score higher than males on items 2 and 5, despite the same location on the latent trait. Opposite results were found for item 6, with males scoring higher than females. Parallel ICCs for males and females indicate evidence of uniform DIF (Fig. 3). For the other person factors available, none of the items displayed DIF when the sample size was adjusted. When data from lower and upper secondary schools were analysed separately, similar results were found based on data from lower secondary schools, whereas only items 2 and 6 displayed gender DIF in data from upper secondary schools. Reducing sample size to 900 no items displayed DIF when it comes to school level or grade.

Gender DIF was resolved by first splitting item 6 (highest F-value) into two separate items, one for males and one for females (Table 4). After item 6 was split the PSI remained approximately the same (0.826). Given the adjusted sample size of 900, item 2, but not item 5, still displayed significant DIF. The same procedure was repeated for item 2, which did not affect the PSI. When items 2 and 6 were split, none of the other items displayed DIF.

**Table 4:** Resolving gender differential item functioning for selected items of Hopkins Symptoms Checklist-10

*n* = 900^#^	Original 10 items	Resolving item 6	Resolving items 2 and 6
Males	− 2.029	− 2.031	− 2.108
Females	− 0.937	− 0.863	− 0.963
Difference between males and females	1.092	1.168	1.145
Total χ^2^ (df), *p*	114 (40), *p* < 0.001	104 (44),* p* < 0.001	104 (48),* p* < 0.001
PSI	0.823	0.826	0.824

^#^Results based on a reduced sample applying the amend sample size function in RUMM2030Plus

When items 2 and 6 were resolved, the difference between the two genders was found to be 0.053 logit bigger than the magnitude of 1.092 in the original set of 10 items, a change of 4.9%. When only item 6 was resolved, the difference between the two genders was 0.076 logit bigger than the magnitude of 1.092 in the original set of 10 items. When item 2 was excluded, a PSI of 0.813 and a Cronbach’s alpha of 0.905 were found. Excluding items 2 and 6 resulted in a PSI of 0.795 and a Cronbach’s alpha of 0.90.

## Discussion

HSCL-10 shows satisfactory psychometric properties at an overall level. It was found to be unidimensional and to have acceptable reliability. However, HSCL-10 shows some weaknesses at a finer level related to response dependency and DIF.

### Targeting

The results indicate that HSCL-10 is somewhat out of target, meaning that adolescents may have better psychological health than the instrument can measure. This was expected because this instrument was originally developed for clinical purposes but was applied in this study on a healthy population. These results are in line with those of Kleppang and Hagquist [14] who nevertheless found that the instrument was even more off target. However, their study was based on data from 2001 and 2009. Since psychological distress has increased among adolescents over the last decade, the instrument is expected to be better targeted at today’s adolescents. In line with the results of Kleppang and Hagquist [14], the results indicate better targeting for females than for males, which might be due to females reporting more psychological complaints than males [6, 32].

Targeting is not considered a problem for this instrument if the intention is to measure psychological distress. However, if the intention is to measure the broader concept of mental health, questions assessing better, or positive mental health should be included. Bad targeting might imply decreased reliability [23], meaning that reliability can be strengthened if the instrument is applied to clinical samples.

### Dimensionality and response dependency

Despite consisting of items intending to measure both symptoms of depression and anxiety, HSCL-10 was found to be unidimensional, although multi-dimensionality was expected from theoretical and medical perspectives. However, depression often co-occurs with anxiety in adolescents [33, 34]. Therefore, when measuring symptoms of depression in adolescents, indicators of anxiety should probably be included.

Contrary to the findings of Kleppang and Hagquist [14], response dependency was found between items 1 and 2, indicating the items to collect redundant information. Several items over-discriminated, including item 2, which might strengthen the evidence of dependency [22]. As revealing response dependency may be dependent on the number of items, more item pairs are expected to show response dependency from more extended versions of the HSCL. As items 1 and 2 can be deemed as collecting redundant information, item 2 can be excluded as this also over-discriminates and displays DIF. A small decrease in PSI and Cronbach’s alpha was found when item 2 was excluded, which may strengthen the hypothesis that response dependency inflates reliability indexes. However, excluding item 2, which pertains to anxiety, creates a conceptually unbalanced construct. Response dependency may also result from translation. In Norwegian, ‘scared’ and ‘fear’ translate to one word close to ‘afraid’. Adolescents may not distinguish between being suddenly scared and feeling fearful. Therefore, the presence of response dependency should be investigated in other languages or translations of HSCL-10.

### Item fit and differential item functioning

In line with Kleppang and Hagquist [14], we found that item 6 under-discriminated. Under-discriminating items tends to measure something else not correlated with the latent trait. Sleep problems and sleeplessness may be related to psychological distress. Schmalbach et al. [11] found a moderately high correlation between the HSCL and the Jenkins Sleep Scale. However, there might be other reasons for sleep problems than psychological distress, such as gaming and being active on social media late at night. The reason for the under-discrimination of this item might also be due to a translation error. The original wording of this item is ‘difficulties in falling asleep or staying asleep’. However, in Norwegian, this translates to ‘sleep problems’, which may be regarded as quite imprecise.

Item 6 also displayed gender DIF, in line with a previous study [14]. In our case, the source of DIF might be explained by gender differences in sleep problems. Social jetlag, sleep deficiency [35, 36] and poor sleep quality [37] have been found to affect females more than males.

Deleting an item may improve the model fit and retain the invariance, but an important aspect of psychological distress may be lost, which may impact the scale’s validity and reliability [28]. When measurement is constructed, items are selected given their relevance as well as representativeness [38]. When resolving an item, the aspect is retained. However, resolving the item bring that the measurement is not invariant among genders, as it will have different difficulties in males and females [28]. To decide whether to resolve DIF additional information about the sources of DIF may be required if the concept in question is not clearly defined [30].

Our results also showed that real DIF in items 2 and 6 affects the person measurement. However, only minor changes in group differences were observed.

Hence, whether an item should be excluded or resolved may be a trade-off between model fit and invariance. If the source of DIF is irrelevant to the variable, such as poor translation, resolving may be justified. When the source of DIF is relevant to the variable, it is not recommended to resolve the DIF as this might worsen the validity [28, 30]. When comparing psychological distress scores across genders, resolving the DIF will have the same result as deleting the item. However, regardless of the source of DIF, invariance should take precedence over fit and the item should not be resolved [30].

The same consideration described above for ‘sleeplessness’ should be adopted for item 2 (‘felt constant fear and anxiety’). The source of DIF for this item may be the result of females recognising the content differently from males. However, the source of DIF is unknown, and further research is encouraged to reveal potential sources of DIF for this item.

While resolving items 2 and 6, item 5 did not display DIF. Hence, the DIF for item 5 can be considered artificial [29, 30].

### Strengths and limitations

This study comprises a large sample size, covers data from the whole country and provides an updated description of adolescents’ responses to HSCL-10. Items were selected from different parts of the questionnaire, some mandatory and some optional, which may influence the response pattern. We also found a discrepancy between the Norwegian wording and the original version of HSCL-10. Our analyses were based on self-reported data. Hence, there is a risk for potential response bias (e.g. social desirability), such as presenting favourable images of themselves. However, considering the large sample size of this study and that the questionnaire was fulfilled anonymously the potential random errors might be minimized [39]*.*

The aim of our study was to assess the psychometric properties for measuring psychological distress in a general population of adolescents. As the HSCL-10 originally was developed for clinical purposes, assessing its clinimetric properties could also be appropriate. However, psychometric and clinimetric assessments could to a large extent be considered to overlap [40, 41]. Nevertheless, future studies should assess the instrument's utility and scalability in clinical practice [42]. Information about respondents’ health status was not available in our study. Hence, future studies should also assess to what extent the items are invariant for people with varying degrees of psychological distress and how well the items discriminate between individuals with different mental health conditions.

## Conclusion

HSCL-10 seems to be a suitable instrument for measuring psychological distress in adolescents. The potential weaknesses discussed here may be related to the imprecise translations of its items. We therefore suggest revising the wording of the Norwegian version to make it more in line with the original version. The instrument’s psychometric properties should also be assessed in other languages. To evaluate its properties in clinical practice clinimetric assessment could also be appropriate. HSCL-10 assesses psychological distress and is not a measure for general mental health. It is important that research employing this instrument uses the right concept. Mental health definitions are considered wider than those of psychological distress.

## Data Availability

Availability of data and materials in the Ungdata surveys are included in a national database administered by Norwegian Social Research (NOVA). Data is available for research purposes upon application. Information on the questionnaires can also be found from the web page (in Norwegian) (http://ungdata.no/).

## Abbreviations

DIF: Differential item functioning
HSCL-10: Hopkins Symptom Checklist-10
ICC: Item-characteristic curve
PSI: Person Separation Index
RMT: Rasch measurement theory
